# Prevalence of Latent Tuberculosis Infection (LTBI) in Mexican Patients With Rheumatoid Arthritis (RA)

**DOI:** 10.7759/cureus.39743

**Published:** 2023-05-30

**Authors:** Abel Eduardo Zavala del Ángel, Jaime Morales-Romero, Roberto Zenteno-Cuevas, José Antonio Enciso Moreno, María del Pilar Mata Miranda, Jorge Luis Martínez Zapata, Clara Luz Sampieri Ramírez, María Gabriela Nachón García, María Sobeida Leticia Blázquez Morales, María Teresa Álvarez-Bañuelos, José Artemio Cruz López, Verónica Patricia Demeneghi-Marini, Laura González-López, Jorge Iván Gámez-Nava

**Affiliations:** 1 Health Sciences Institute, Universidad Veracruzana, Xalapa, MEX; 2 Public Health Institute, Universidad Veracruzana, Xalapa, MEX; 3 Biomedical Sciences, Instituto Mexicano del Seguro Social, Zacatecas, MEX; 4 Family Medicine Unit No. 66, Instituto Mexicano del Seguro Social, Xalapa, MEX; 5 Rheumatology and Internal Medicine, Instituto Mexicano del Seguro Social, Xalapa, MEX; 6 Public Health Coordination, Regional Decentralized Administrative Operation Organ Veracruz-Norte, Instituto Mexicano del Seguro Social, Xalapa, MEX; 7 University Center of Health Sciences, Universidad de Guadalajara, Guadalajara, MEX

**Keywords:** risk factors, interferon-gamma release tests, prevalence, igra, rheumatoid arthriitis, latent tuberculosis infection

## Abstract

Introduction: Patients with rheumatoid arthritis (RA) are at increased risk of developing tuberculosis, and even more so if they receive biological agents. In Mexico, the prevalence of latent tuberculosis infection (LTBI) in RA diagnosed by interferon-gamma release assay (IGRA) is largely unknown. The objective was to determine LTBI prevalence and the associated risk factors in rheumatoid arthritis patients.

Methods: A cross-sectional study was performed comprising 82 patients with RA who attended the rheumatology service at a second-level hospital. Demographic characteristics, comorbidity, Bacillus Calmette-Guerin (BCG) vaccination and smoking history, type of treatment, disease activity and functional capacity were investigated. The Disease Activity Score 28 and the Health Assessment Questionnaire-Disability Index were applied for the estimate of RA activity and functional capacity. Further information was compiled from the electronic medical records and personal interviews. LTBI was determined by QuantiFERON TB Gold Plus (QIAGEN, Germantown, USA).

Results: Prevalence of LTBI was 14% (95% confidence interval (CI): 8.6% to 23.9%). Factors associated with LTBI were history of smoking (odds ratio (OR) = 6.63 95% CI 1.01 to 43.3) and disability score (OR = 7.19 95%CI 1.41 to 36.6).

Conclusions: The prevalence of LTBI in Mexican patients with RA was 14%. Our results suggest prevention of smoking and functional incapacity could reduce the risk of LTBI. Further research could endorse our results.

## Introduction

Rheumatoid arthritis (RA) is a chronic autoimmune disease, characterized by multiple joint inflammation, that commonly causes destructive bone erosion [1]. In recent decades in Mexico, RA prevalence is estimated to be about 1.6%, with variations across geographical locations [2]. Patients with RA require constant monitoring in order to reduce rheumatic disease progression [3]. However, in patients with RA, a latent tuberculosis infection (LTBI) must be excluded prior to the initiation of biological disease-modifying anti-rheumatic drugs (DMARDs) since this can reactivate the infection into active tuberculosis [4-6].

LTBI is characterized by inactive *M. tuberculosis* in the lung parenchyma or other organs, with a persistent immune response to mycobacterium antigens but without producing symptoms of tuberculosis disease. Treatment with anti-tumor necrosis factor (TNF) agents in LTBI patients can favor granuloma development with subsequent growth of the mycobacterium [4].

As a strategy for tuberculosis control, the World Health Organization has established the detection of LTBI in high-risk populations, such as patients with RA, and mainly those that are being treated with anti-TNF agents [7]. It is estimated that up to a fourth of the world’s population has LTBI [8]. In Mexico, the prevalence of LTBI is unknown, but it is estimated to be high when we consider that this country reported an incidence of pulmonary tuberculosis of 15.6 per 100,000 inhabitants in 2019 [9]. One of the difficulties in identifying LTBI by tuberculin skin tests (TST) in Mexico is the high vaccination coverage (>98%) with Bacillus Calmette-Guerin (BCG) among the population [10]. This acts to increase the incidence of false positives in the TST, presenting a low specificity (up to 49%) when applied to patients undergoing biological therapy [11,12]. However, the interferon-gamma release assay (IGRA) is a diagnostic test for LTBI with advantages over TST, since a history of BCG vaccination does not influence its result, nor does it produce false positives with mycobacteria from other species [13].

In addition, discrepancies in the prevalence of LTBI have been described as dependent on the detection method. Generally, the frequency of positive cases is higher for TST than for IGRA in the same sample of subjects [14]. The factors associated with this infection are also not well characterized [15,16]. The aim of this study is to determine the prevalence of LTBI and associated actors in Mexican adults with RA attending a second-level care hospital.

## Materials and methods

This cross-sectional study was conducted from February 28, 2019, to March 30, 2020. 105 patients with a diagnosis of established RA, who consecutively attended an out-patient rheumatology clinic in a secondary care center facility in Xalapa, Mexico, were invited to participate. For inclusion, the patients had to have an RA diagnosis established by a rheumatologist that met the 1987 American College of Rheumatology and the 2010 European League Against Rheumatism criteria and had to sign a voluntary agreement to participate in the study [17-19]. Subjects under 18 years of age, those who were pregnant or breastfeeding and patients with a history of HIV infection or hepatitis C were excluded. Demographic and clinical information, RA treatment and other relevant backgrounds were collected through interview. A structured clinical chart review of the electronic medical record for each patient was developed. This review identified important RA characteristics, laboratory variables and treatments.

LTBI diagnosis

Diagnosis of LTBI was established through QuantiFERON TB Gold Plus (QIAGEN, Germantown, USA) instructions: 5 ml of venous blood was extracted from the arm into tubes with lithium heparin, and 1 ml of this hematic sample was exported to each QuantiFERON kit tube. After incubation for 20 hours, 200 μl aliquots were prepared and stored at -60°C for subsequent shipment to the Medical Research Unit of Zacatecas, where reagents were reconstituted for reading using the enzyme-linked immunoassay (ELISA) technique. Preanalytical and analytical techniques were performed by trained professionals and following the manufacturer's indications [20].

Evaluating possible risk factors

Disease activity was estimated according to the Disease Activity Score 28 (DAS 28) questionnaire. The results were categorized according to the following criteria: ≥ 5.1 (high), ≥ 3.2 to < 5.1 (moderate), ≥ 2.6 to < 3.2 (low) and < 2.6 (remission) [21]. Physical disability was determined according to The Health Assessment Questionnaire Disability Index (HAQ-DI), in which all patients with a score value of ≥ 0.6 were considered to present a physical disability [22]. History of BCG vaccination was determined in the personal interview and confirmed by verifying the presence of the typical scar on the arm. Comorbidity was determined through the electronic medical record, particularly that of type 2 diabetes mellitus and arterial hypertension. Smoking history was categorized as “never smoked,” “history of smoking but not currently,” and “current smoker” [23]. Current RA treatment with synthetic disease-modifying anti-rheumatic drugs (synthetic-DMARDs), biological-DMARDs, non-steroid anti-inflammatory agents (NSAIDs) or corticosteroids was determined by the interview and confirmed through review of the electronic records.

Statistical analysis

The quantitative variables were expressed as means ± standard deviations (or medians and ranges if they followed a non-parametric distribution). The qualitative variables were expressed as frequencies and percentages and compared using the chi-square or Fisher's exact test. The prevalence of LTBI and its 95% confidence intervals was calculated using the Wilson score formula. We performed a univariate analysis to calculate the variables associated with the risk of LTBI, identifying the crude odds ratios (ORs) and their 95% confidence intervals (CIs). In the multivariate binary logistic regression analysis, we identified the risk factors for LTBI adjusted for potential confounders. In these models, the dependent variable was the presence of LTBI, and the independent covariates that were tested were physical disability, smoking history, RA disease duration, positive rheumatoid factor, current use of biological agents, use of corticosteroids, antecedents of BCG vaccination, antecedents of contact with patients with tuberculosis, sex (males) and level of formal education. The Enter method and forward stepwise methods were used to introduce the variables into the model. ORs and their 95% CIs were estimated. Data analysis was performed using Microsoft Excel programs for Windows and Mac, version 16.48; Epi InfoTM version 7.2.4.0 (Centers for Disease Control and Prevention, USA); OpenEpi: Open-Source Epidemiologic Statistics for Public Health, Version 3.01; Epidat: Program for Epidemiological Data Analysis, version 4.2 (July 2016) and IBM SPSS Statistics for Mac (IBM, Version 23.0. Armonk, USA).

Ethics

This research was approved by the National Committee for Scientific Research of the Mexican Institute of Social Security (R-2018-785-113), the Research Committee and the Research Ethics Committee of the Institute of Health Sciences of the University of Veracruz. Furthermore, all patients voluntarily agreed to participate by an informed consent form. All the identified LTBI cases were referred to epidemiology for medical attention.

## Results

Of 105 patients who met the inclusion criteria and were invited to participate in the study, 21 did not agree to participate and two had an indeterminate result in the IGRA. The definitive sample therefore consisted of n = 82.

Description of the population

Women were more frequent than men (93%), mean age was almost 53 years (± 10.8), nearly 79% had formal education greater than primary level and 31% reported previous or current smoking. In addition, 45% presented some comorbidity other than LTBI, the most frequent being arterial hypertension, followed by type 2 diabetes mellitus. Regarding the characteristics of RA, 56% had a disease duration of less than ten years, 88% had a positive rheumatoid factor, 96.3% had high or moderate RA activity, and 40.2% reported some degree of physical disability (Table 1). The average HAQ-DI score was 0.57 ± 0.5, with 2.2 being the maximum value. The treatment most used by the patients was synthetic-DMARDs (96.3%), the most frequent of which were methotrexate, leflunomide and chloroquine (75.6%, 63.4%, and 58.5%, respectively). Biological therapy was received in 43% of subjects, with certolizumab pegol being the most commonly used, followed by rituximab (14.6% and 12.2%, respectively). Finally, regarding the history related to tuberculosis, 89% were vaccinated with BCG and 17% reported having had previous contact with a case of tuberculosis, an average of 17.3 years previously.

**Table 1:** Clinical characteristics of the study sample P_25_: 25th percentile; P_50_; 50th percentile; P_75_; 75th percentile; RA: Rheumatoid arthritis; BCG: Bacillus Calmette-Guerin; NSAIDs: Non-steroidal anti-inflammatory drugs; DMARDs: Disease-modifying anti-rheumatic drugs. Physical disability was evaluated through Health Assessment Questionnaire-Disability Index (HAQ-DI ≥ 0.6). Clinical activity of RA was evaluated through disease activity score (DAS28).

Clinical characteristics	
Variables	n = 82
Sex (female), n (%)	76 (92.7)
Age (years) mean ± SD	52.7 ± 10.8
Level of education ≤ elementary school n (%)	17 (20.7)
RA duration (years), P_50_ (P_25_, P_75_)	9.5 (4.7,16.5)
RA duration more than 10 years, n (%)	36 (43.9)
Positive rheumatoid factor, n (%)	72 (87.8)
BCG vaccine, n (%)	73 (89.0)
Previous contact with tuberculosis patient, n (%)	14 (17.1)
Never smoked, n (%)	57 (69.5)
Current or former smoker, n (%)	25 (30.5)
Physical disability, n (%)	33 (40.2)
Arterial hypertension, n (%)	22 (26.8)
Diabetes mellitus type 2, n (%)	11 (13.4)
High disease activity, n (%)	51 (63.0)

LTBI prevalence

Of the 84 patients included initially, 12 were reactive to the IGRA, 70 were non-reactive, and two were indeterminate. Therefore, the ultimate sample size was n = 82 patients. LTBI prevalence was 14.6% (95%CI 8.6% to 23.9%). Prevalence of LTBI was higher in the following variables: 1) people > 50 years (15.1% 95%CI 7.9% to 27.1%); 2) males (33.3%, 95%CI 9.7% to 70.0%); 3) those with no spouse or partner (19.4%, 95%CI 9.2% to 36.3%); 4) those with low level of education (≤ elementary school) (23.5% 95%CI 9.6% to 47.3%); 5) those with a history of smoking (28.0% 95%CI 14.3% to 47.6%); 6) those with positive rheumatoid factor (15.3%, 95%CI 8.8% to 25.3%); 7) those not vaccinated with BCG (22.2%, 95%CI 6.3% to 54.7%); 8) those with active disease (15.2%, 95%CI 8.9% to 24.7%); 9) those with physical disability (6.1%, 95%CI 2.1% to 16.5%); and 10) those with arterial hypertension (18.2%, 95%CI 7.3% to 38.5%). Prevalence of LTBI, history of smoking and physical disability in patients with the three conditions simultaneously was 4.9 % (CI95% 1.9 % to 11.9 %) (data not shown in tables).

Factors associated with LTBI prevalence

In the univariate analysis, the disability index (OR ≈ 5.8, p = 0.01) and smoking history (OR ≈ 4, p = 0.02) separately presented a statistically significant association with LTBI. In contrast, there was no significant association with age (> 50 years), sex (men), having a spouse or partner, level of education, duration of the disease (> 10 years), previous contact with a tuberculosis patient, BCG vaccination history, comorbidity (diabetes or hypertension), clinical disease activity, positive rheumatoid factor or treatment (Table 2).

**Table 2:** Univariate analysis of possible LTBI risk factors LTBI: Latent tuberculosis infection; RA: Rheumatoid arthritis; BCG: Bacillus Calmette-Guerin; NSAIDs: Nonsteroidal anti-inflammatory drugs; DMARDs: Disease-modifying anti-rheumatic drugs. NA: Not applicable. Proportions were compared by chi-square test.

Variables					
n (%)	LTBI (n = 12)	Non-LTBI (n = 70)	OR	95%CI	*p*
Age (> 50 years)	8 (66.7)	45 (64.3)	1.11	0.3 to 4.1	0.99
Sex (men)	2 (16.7)	4 (5.7)	3.3	0.5 to 20.4	0.21
With a spouse or partner	6 (50.0)	45 (64.3)	0.56	0.2 to 1.9	0.36
≤ Elementary school	4 (33.3)	13 (18.6)	2.2	0.6 to 8.4	0.26
RA duration > 10 years	5 (41.7)	31 (42.9)	0.90	0.3 to 3.1	0.86
Previous contact with a tuberculosis patient	11 (91.7)	61 (87.1)	0.4	0.1 to 3.7	0.68
History of BCG vaccine	10 (83.3)	63 (90.0)	0.56	0.1 to 3.1	0.61
Type 2 diabetes mellitus	1 (8.3)	10 (14.3)	0.55	0.1 to 4.7	0.58
Arterial hypertension	4 (33.3)	18 (25.7)	1.44	0.4 to 5.4	0.58
Disease activity	12 (100)	66 (95.7)	NA	NA	0.46
Physical disability	9 (75.0)	24 (34.3)	5.75	1.4 to 23.2	0.01
History of smoking	7 (58.3)	18 (22.0)	4.04	1.1 to 14.3	0.023
Positive rheumatoid factor	11 (91.7)	61 (87.1)	1.62	0.2 to 14.1	0.99
NSAIDs	11 (91.6)	64 (91.4)	1.03	0.1 to 9.4	0.99
Synthetic-DMARDs	12 (100)	67 (95.7)	NA	NA	0.99
Biological-DMARDs	5 (41.7)	30 (42.8)	0.95	0.3 to 3.3	0.94
Steroids	11 (91.7)	59 (84.3)	2.05	0.2 to 17.5	0.68

Association between LTBI with physical disability and smoking history was found in the multivariate analysis. In model 1, only the two previous variables were introduced. In model 2, the duration of the disease, the positive rheumatoid factor, the biological therapy and the steroid treatment was added. In model 3, history of BCG vaccination and previous contact with a patient with tuberculosis were also entered. Finally, in model 4, adjustment was made for age and sex. In all models, physical disability and smoking history showed a strong association with LTBI. In contrast, the rest of the variables introduced in the four models did not show a significant association (Table 3).

**Table 3:** Multivariate logistic regression analysis of possible LTBI risk factors Physical disability: HAQ-DI ≥ 0.6 LTBI: Latent tuberculosis infection; HAQ-DI: Health Assessment Questionnaire-Disability Index; RA: Rheumatoid arthritis; RF: Rheumatoid factor; DMARDs: Disease-modifying anti-rheumatic drugs; BCG: Bacillus Calmette-Guerin. All variables were introduced as categorical variables, except for RA duration.

Multivariate models comparison												
	Model 1			Model 2			Model 3			Model 4		
Variable	OR	95%CI	p	OR	95%CI	p	OR	95%CI	p	OR	95%CI	p
Physical disability	6.7	1.5 to 29.0	0.011	6.5	1.4 to 29.4	0.016	8.1	1.7 to 38.1	0.009	7.1	1.4 to 36.6	0.018
Smoking history	4.9	1.2 to 19.1	0.024	6.7	1.5 to 30.5	0.014	5.8	1.2 to 28.5	0.03	6.6	1.0 to 43.3	0.048
RA duration (years)				1.03	0.97 to 1.1	0.38	1.03	0.96 to 1.1	0.40	1.02	1.0 to 1.1	0.60
Positive RF				3.1	0.3 to 37.1	0.37	2.4	0.2 to 30.8	0.51	1.5	0.1 to 21.6	0.79
Biological-DMARDs				0.8	0.2 to 3.5	0.78	0.9	0.2 to 3.9	0.88	0.9	0.2 to 4.43	0.94
Treatment with steroids				1.8	0.2 to 18.9	0.65	1.8	0.2 to 20.8	0.62	3.1	0.1 to 75.6	0.48
BCG vaccine							0.8	0.1 to 7.9	0.81	1.6	0.1 to 20.9	0.73
Previous tuberculosis contact							0.3	0.03 to 2.5	0.24	0.2	0.01 to 2.6	0.20
Sex (men)										6.0	0.4 to 102	0.21
≤ Elementary school										3.0	0.4 to 23.7	0.31

## Discussion

In this study, the prevalence of LTBI in patients with RA was 7% to 22%. Compared with developed countries, Mexico has important risk factors for tuberculosis infection as it has high rates of poverty and malnutrition, a low average education level, and a high proportion of people with limited access to health services. LTBI is particularly important in RA because these patients tend be treated with biological agents, particularly anti-TNF agents that can reactivate tuberculosis infection [16].

The prevalence of LTBI in rheumatic diseases measured through the TST is usually overestimated so it is recommended to be diagnosed by IGRA. For example, in research conducted in New York, USA, the prevalence of LTBI by TST was 31.8% while that by IGRA was 17.4%, similar to our study [14]. Another high prevalence in rheumatic diseases using TST was found in South Korea (37%) [24]. In Latin America, Brazil is one of the countries with the highest prevalence reported of LTBI in RA patients undergoing anti-TNF treatments (44.2%) [15]. Instead, in developed countries such as United Kingdom, a study using IGRA in patients on biological-DMARDs identified only 10% with LTBI, a lower prevalence compared to that found in our study [25].

The prevalence of LTBI in RA in Mexico, measured through the TST, ranges from 21.4% to 33.5% (cut-off point: an induration ≥ 5 mm) [26,27]. However, this prevalence could have been overestimated due to the high BCG vaccination coverage in Mexico or previous exposure to non-tuberculous mycobacteria [10,12]. False-positive results in BCG vaccinated subjects act to produce an apparently higher prevalence than that determined by IGRA [28]. Although concordance between the TST and IGRA results is usually greater in those countries with a low tuberculosis incidence or low BCG vaccination coverage, detection by IGRA is recommended in regions with this type of immunization, since this method has greater specificity than the TST [13,29,30]. Unfortunately, the higher cost and need for specialized equipment and personnel act to restrict its use [28,30].

Diabetes can also contribute to the frequency of LTBI. The Mexican state of Veracruz, the site of this study, has a high incidence of tuberculosis and a high prevalence of diabetes nationwide [31,32]. In Mexico, it has been found that more than half of the subjects with diabetes also present LTBI [33]. Contrary to the above, although LTBI was more frequent in subjects without diabetes in our study, the difference shown was not statistically significant. The cross-sectional nature of the design and the size of the sample studied could explain this result.

In our study, two factors associated with LTBI were identified: smoking and impaired function for activities of daily living. Regarding smoking, results of studies by other authors coincide with ours [34]. Previous or current smoking produces the development of active or latent tuberculosis since it reduces the capacity for clearance of secretions in the lung mucosa and the phagocytic capacity of alveolar macrophages [35,36]. This is important in a country as Mexico where 24.6% of people aged 18 or over smoke [37].

Concerning functional capacity impairment, a HAQ-DI score > 0.6 showed a higher risk of LTBI in our multivariate analysis, which could result from an indirect relationship mediated by the activity of the disease or the treatment [16,38]. However, no significant association was found between LTBI and disease activity or the use of biological-DMARDs or glucocorticoids when analyzing these variables. As an explanation, it should be considered that practically all (93.4%) of the subjects that had an active disease in our study had LTBI. On the other hand, regarding treatment, although the LTBI group received some glucocorticoid more frequently (91.7% vs 84.3%), the differences found were not significant. Likewise, the use of biological-DMARDs in patients with and without LTBI was similar. The lack of association of LTBI with disease activity and treatment contrasts with the results of other authors who indicate a significant association [16,38]. Again, the cross-sectional nature of the design and the sample size could explain these findings, and, therefore, we recommend conducting further studies in this regard.

Although some authors indicate a significant association between age and sex with LTBI in the general population, our study did not demonstrate this due to most participants were women (92.9%), and only two men had LTBI [34]. We also found no association between low educational levels and the prevalence of LTBI.

Can BCG vaccination have a protective effect on LTBI? In Mexico, most of the child population is vaccinated with BCG (98%) [10]. Our multivariate analysis yielded contrasting and non-significant results according to the adjustment model. We therefore recommend further studies to elucidate the relationship between BCG vaccination and LTBI.

When there is a previous exposure to *M. tuberculosis*, patients with rheumatic diseases have a higher risk of developing tuberculosis [15]. However, our study showed a non-significant inverse association in patients with prior contact. It is important to note that this previous exposure was determined through the patient's self-report, so it could represent an underestimation of frequency, since there is a low detection rate of patients with tuberculosis in Mexico [39].

Limitations and strengths of the study

One of the study's main limitations was the interruption in access to patients that occurred due to the SARS-CoV-2 epidemic in Mexico. The lack of statistical significance in some of the explored associations in the present investigation may be the result of the relatively small sample size. A third limitation was the lack of comparison of the IGRA results with those of the TST. On the other hand, the use of IGRA and the modeling carried out in the multivariate analysis in the search for associations constitute the study's main strengths.

## Conclusions

In conclusion, the present investigation demonstrates a high prevalence of LTBI in RA patients from an area of southeastern Mexico. Our findings suggest it is advisable to develop a permanent LTBI screening program in Mexican RA patients, in particular on those exposed to smoking or functional impairment since both proved to be risk factors for the latent infection. Therefore, LTBI diagnosis based on risk factors might contribute to the decline of tuberculosis reactivation since all infected patients need to be refered for early chemoprophylactic treatment.
